# Global real-world experiences with pembrolizumab in advanced urothelial carcinoma after platinum-based chemotherapy: the ARON-2 study

**DOI:** 10.1007/s00262-024-03682-w

**Published:** 2024-04-18

**Authors:** Francesco Massari, Matteo Santoni, Hideki Takeshita, Yohei Okada, Jose Carlos Tapia, Umberto Basso, Marco Maruzzo, Sarah Scagliarini, Thomas Büttner, Giuseppe Fornarini, Zin W. Myint, Luca Galli, Vinicius Carrera Souza, Renate Pichler, Ugo De Giorgi, Nathalia Gandur, Elaine T. Lam, Danielle Gilbert, Lazar Popovic, Enrique Grande, Giulia Mammone, Rossana Berardi, Simon J. Crabb, Robert Kemp, Javier Molina-Cerrillo, Marcelo Freitas, Murilo Luz, Roberto Iacovelli, Fabio Calabrò, Deniz Tural, Francesco Atzori, Zsófia Küronya, Rita Chiari, Saul Campos, Orazio Caffo, André P. Fay, Jakub Kucharz, Paolo Andrea Zucali, José Augusto Rinck, Annalisa Zeppellini, Diogo Assed Bastos, Gaetano Aurilio, Augusto Mota, Karine Trindade, Cinzia Ortega, Juan Pablo Sade, Mimma Rizzo, Ondřej Fiala, Nuno Vau, Patrizia Giannatempo, Allan Barillas, Fernando Sabino M. Monteiro, Breno Dauster, Alessia Mennitto, Lucas Nogueira, Roni de Carvalho Fernandes, Emmanuel Seront, Luís Garcia Aceituno, Francesco Grillone, Hernan Javier Cutuli, Mauricio Fernandez, Maria Bassanelli, Ray Manneh Kopp, Giandomenico Roviello, Halima Abahssain, Giuseppe Procopio, Michele Milella, Jindrich Kopecky, Angelo Martignetti, Carlo Messina, Manuel Caitano, Eva Inman, Ravindran Kanesvaran, Daniel Herchhorn, Daniele Santini, Aristotelis Bamias, Renato Bisonni, Alessandra Mosca, Franco Morelli, Fernando Maluf, Andrey Soares, Fernando Nunes, Alvaro Pinto, Anca Zgura, Lorena Incorvaia, Jawaher Ansari, Ignacio Ortego Zabalza, Johannes Landmesser, Alessandro Rizzo, Veronica Mollica, Andrea Marchetti, Matteo Rosellini, Giulia Sorgentoni, Nicola Battelli, Sebastiano Buti, Camillo Porta, Joaquim Bellmunt

**Affiliations:** 1 Medical Oncology, IRCCS Azienda Ospedaliero-Universitaria di Bologna, Bologna, Italy; 2 Oncology Unit, Macerata Hospital, via Santa Lucia 2, 62100 Macerata, Italy; 3 Department of Urology, Saitama Medical Center, Saitama Medical University, Saitama, Japan; 4 Department of Medical Oncology, Institut d’Investigació Biomèdica Sant Pau, Hospital de la Santa Creu i Sant Pau, Barcelona, Spain; 5 Medical Oncology 1 Unit, Department of Oncology, Istituto Oncologico Veneto IOV IRCCS, 35128 Padova, Italy; 6 UOC di Oncologia, Azienda Ospedaliera di Rilievo Nazionale Cardarelli di Napoli, Naples, Italy; 7 Department of Urology, University Hospital Bonn (UKB), 53127 Bonn, Germany; 8 IRCCS Ospedale Policlinico San Martino, Genoa, Italy; 9 Markey Cancer Center, University of Kentucky, Lexington, KY 40536-0293 USA; 10 Oncology Unit 2, University Hospital of Pisa, 56126 Pisa, Italy; 11 Hospital São Rafael Oncologia D’Or, Salvador, BA Brazil; 12 Latin American Cooperative Oncology Group – LACOG, Porto Alegre, Brazil; 13 Department of Urology, Medical University of Innsbruck, Anichstrasse 35, 6020 Innsbruck, Austria; 14 Department of Medical Oncology, IRCCS Istituto Romagnolo per lo Studio dei Tumori (IRST) “Dino Amadori”, Meldola, Italy; 15 Hospital Angel Roffo, Buenos Aires, CABA Argentina; 16 University of Colorado Anschutz Medical Campus, Aurora, CO USA; 17 Oncology Institute of Vojvodina, Faculty of Medicine, University Novi Sad, Novi Sad, Serbia; 18 Department of Medical Oncology, MD Anderson Cancer Center Madrid, Madrid, Spain; 19 Department of Radiological, Oncological and Anatomo-Pathological Science, “Sapienza” University of Rome, Viale Regina Elena 324, 00185 Rome, Italy; 20 Department of Medical Oncology, Università Politecnica Delle Marche, AOU Ospedali Riuniti Delle Marche, Ancona, Italy; 21 Southampton Experimental Cancer Medicine Centre, University of Southampton, Southampton, UK; 22 Department of Medical Oncology, Hospital Ramón y Cajal, Madrid, Spain; 23 Centro de Pesquisas Oncológicas - CEPON, Florianópolis, SC Brazil; 24 Hospital Erasto Gaertner, Curitiba, PR Brazil; 25 Oncologia Medica, Fondazione Policlinico Universitario Agostino Gemelli IRCCS, Rome, Italy; 26 Department of Oncology, San Camillo Forlanini Hospital, Rome, Italy; 27 Department of Medical Oncology, Bakirköy Dr. SadiKonuk Training and Research Hospital, Tevfik Saglam St. No: 11, Zuhuratbaba District, Bakirkoy, Istanbul, Turkey; 28 Unità di Oncologia Medica, Azienda Ospedaliero Universitaria di Cagliari, Cagliari, Italy; 29 Department of Genitourinary Medical Oncology and Clinical Pharmacology, National Institute of Oncology, Budapest, Hungary; 30 UOC Oncologia, Azienda Ospedaliera Ospedali Riuniti Marche Nord, Fano, Italy; 31 Centro Oncologico Estatal “Dr José Luis Barrera Franco” del ISSEMYM, Toluca de Lerdo, Mexico; 32 Medical Oncology Unit, Santa Chiara Hospital, Trento, Italy; 33 Pontificia Universidade Católica do Rio Grande do Sul - PUCRS, Porto Alegre, RS Brazil; 34 Department of Uro-Oncology, Maria Sklodowska-Curie National Research Institute of Oncology Warsaw, Warsaw, Poland; 35 Department of Oncology, IRCCS Humanitas Research Hospital, Rozzano - Milan, Italy; 36 Department of Biomedical Sciences, Humanitas University, Pieve Emanuele, Milan, Italy; 37 Hospital AC Camargo, São Paulo, SP Brazil; 38 Niguarda Cancer Center, Grande Ospedale Metropolitano Niguarda, Milan, Italy; 39 Department of Oncology, Hospital Sírio-Libanês, São Paulo, SP Brazil; 40 Division of Cancer Prevention and Genetics, IEO European Institute of Oncology IRCCS, Milan, Italy; 41 Clínica AMO, Salvador, BA Brazil; 42 Oncologia D’Or, Fortaleza, CE Brazil; 43 ASLCN2 Alba-Bra, Ospedale Michele E Pietro Ferrero, Verduno, CN Italy; 44 Instituto Alexander Fleming, Buenos Aires, CABA Argentina; 45 Division of Medical Oncology, A.O.U. Consorziale Policlinico Di Bari, Piazza G. Cesare 11, 70124 Bari, Italy; 46 Department of Oncology and Radiotherapeutics, Faculty of Medicine, University Hospital in Pilsen, Charles University, Pilsen, Czech Republic; 47 Urologic Oncology, Champalimaud Clinical Center, 1400-038 Lisbon, Portugal; 48 Dipartimento di Oncologia Medica, Fondazione IRCCS Istituto Nazionale Dei Tumori, Milan, Italy; 49 Clinicas Medicas Especializadas NUCARE, Guatemala City, Guatemala; 50 Hospital Sirio-Libanês, Brasília, DF Brazil; 51 Hospital Sao Rafael, Salvador, BA Brazil; 52 Department of Medical Oncology, “Maggiore Della Carità” University Hospital, 28100 Novara, Italy; 53 Universidade Federal de Minas Gerais - UFMG, Belo Horizonte, MG Brazil; 54 Hospital Santa Casa de Sao Paulo, São Paulo, SP Brazil; 55 Department of Medical Oncology, Centre Hospitalier de Jolimont, Haine Saint Paul, Belgium; 56 Clinica Medica Especializada en Oncologia Medica, Guatemala City, Guatemala; 57 UO Oncologia Azienda Ospedaliera Universitaria Renato Dulbecco PO Pugliese Ciaccio Catanzaro, Catanzaro, Italy; 58 Hospital Sirio Libanes, Buenos Aires, CABA Argentina; 59 Fundacion Centro Oncologico de Integracion Regional - COIR, Mendoza, Argentina; 60 Medical Oncology 1-IRCCS Regina Elena National Cancer Institute, Rome, Italy; 61 Clinical Oncology, Sociedad de Oncología y Hematología del Cesar, Valledupar, Colombia; 62 Department of Health Sciences, Section of Clinical Pharmacology and Oncology, University of Florence, Viale Pieraccini 6, 50139 Florence, Italy; 63 Medicine and Pharmacy Faculty, National Institute of Oncology, Medical Oncology Unit, Mohamed V University, Rabat, Morocco; 64 Oncologia Medica, Ospedale Maggiore di Cremona, Cremona, Italy; 65 Section of Oncology, Department of Medicine, University of Verona School of Medicine and Verona University Hospital Trust, Verona, Italy; 66 Department of Clinical Oncology and Radiotherapy, University Hospital Hradec Kralove, Hradec Kralove, Czech Republic; 67 Dipartimento Oncologico USL Sud-Est Toscana-Area Senese, Località Campostaggia S.N.C, 53036 Poggibonsi, Italy; 68 Oncology Unit, A.R.N.A.S. Civico, Palermo, Italy; 69 Hospital do Câncer Porto Dias – Rede Mater Dei de Saúde, Belém, PA Brazil; 70 ONCOR Life Medical Center, Saltillo, Mexico; 71 National Cancer Centre Singapore, Singapore, Singapore; 72 Instituto D’Or de Ensino e Pesquisa, Rio de Janeiro, RJ Brazil; 73 Department of Radiological, Oncological and Pathological Sciences, Policlinico Umberto I, University of Rome, SapienzaRome, Italy; 74 2nd Propaedeutic Department of Internal Medicine, ATTIKON University Hospital, School of Medicine, National and Kapodistrian University of Athens, Athens, Greece; 75 UOC Oncologia Medica, Ospedale A. Murri, Fermo, Italy; 76 Oncology, Candiolo Cancer Institute, IRCCS-FPO, 10060 Turin, Italy; 77 Medical Oncology Unit, Gemelli Molise Hospital, Università Cattolica del Sacro Cuore, Campobasso, Italy; 78 Hospital Beneficencia Portuguesa de São Paulo, São Paulo, SP Brazil; 79 Hospital Israelita Albert Einstein, São Paulo, SP Brazil; 80 Clinica de Oncologia - Clion, Salvador, BA Brazil; 81 Medical Oncology Department, La Paz University Hospital, Madrid, Spain; 82 Department of Oncology-Radiotherapy, Prof. Dr. Alexandru Trestioreanu Institute of Oncology, Carol Davila; University of Medicine and Pharmacy, Bucharest, Romania; 83 Department of Precision Medicine in Medical, Surgical and Critical Care (Me.Pre.C.C.), Section of Medical Oncology, University of Palermo, Via del Vespro 129, 90127 Palermo, Italy; 84 Medical Oncology, Tawam Hospital, Al Ain, United Arab Emirates; 85 Klinik für Urologie, Ratzeburger Allee 160, 23538 Lübeck, Germany; 86 Struttura Semplice Dipartimentale di Oncologia Medica per la Presa in Carico Globale del Paziente Oncologico “Don Tonino Bello”, I.R.C.C.S. Istituto Tumori “Giovanni Paolo II”, Viale Orazio Flacco 65, 70124 Bari, Italy; 87 Medical Oncology Unit, Department of Medicine and Surgery, University Hospital of Parma, University of Parma, Parma, Italy; 88 Chair of Oncology, Interdisciplinary Department of Medicine, University of Bari “Aldo Moro”, Bari, Italy; 89 Dana Farber Cancer Institute, Harvard Medical School, Boston, MA USA

**Keywords:** ARON-2 study, Pembrolizumab, Real-world data, Survival, Tumor response, Urothelial cancer

## Abstract

**Background:**

Immune checkpoint inhibitors have changed previous treatment paradigm of advanced urothelial carcinoma (UC). The ARON-2 study (NCT05290038) aimed to assess the real-world effectiveness of pembrolizumab in patients recurred or progressed after platinum-based chemotherapy.

**Patients and Methods:**

Medical records of patients with documented metastatic UC treated by pembrolizumab as second-line therapy were retrospectively collected from 88 institutions in 23 countries. Patients were assessed for overall survival (OS), progression-free survival (PFS) and overall response rate (ORR). Cox proportional hazards models were adopted to explore the presence of prognostic factors.

**Results:**

In total, 836 patients were included: 544 patients (65%) received pembrolizumab after progression to first-line platinum-based chemotherapy in the metastatic setting (cohort A) and 292 (35%) after recurring within < 12 months since the completion of adjuvant or neoadjuvant chemotherapy (cohort B). The median follow-up time was 15.3 months. The median OS and the ORR were 10.5 months and 31% in the overall study population, 9.1 months and 29% in cohort A and 14.6 months and 37% in cohort B. At multivariate analysis, ECOG-PS ≥ 2, bone metastases, liver metastases and pembrolizumab setting (cohort A vs B) proved to be significantly associated with worst OS and PFS. Stratified by the presence of 0, 1–2 or 3–4 prognostic factors, the median OS was 29.4, 12.5 and 4.1 months (*p* < 0.001), while the median PFS was 12.2, 6.4 and 2.8 months, respectively (*p* < 0.001).

**Conclusions:**

Our study confirms that pembrolizumab is effective in the advanced UC real-world context, showing outcome differences between patients recurred or progressed after platinum-based chemotherapy.

**Supplementary information:**

The online version contains supplementary material available at 10.1007/s00262-024-03682-w.

## Introduction

The World Health Organization (WHO) has globally estimated 573,278 new cases of bladder cancer in 2020 [1]. In the same year, the number of bladder cancer-related deaths has been estimated at 212,536, the 75% of which are in men [1]. Urothelial cancer (UC) is the most frequent histologic variant of tumors of the upper and lower urinary tracts, representing approximately 90% of all new diagnoses [2]. The aggressive behavior of this tumor, which accounts for a dramatically low 5-year survival rate of around 7% [3], has pushed cancer researchers to develop novel therapeutic approaches with the aim of improving the management of UC patients and to exceed the results obtained by chemotherapy in this context [4]. The advent of immune checkpoint inhibitors has, at least in part, changed the previous treatment paradigm of advanced UC patients progressing after platinum-based chemotherapy [5]. The Food and Drugs Administration (FDA) approved pembrolizumab based on the results of the KEYNOTE-045 randomized phase III trial [6]. In this trial, patients were randomized to receive pembrolizumab at a dose of 200 mg every 3 weeks or the investigator's choice of chemotherapy with paclitaxel, docetaxel or vinflunine. Patients progressed after platinum-based chemotherapy or recurred within 12 months since completion of adjuvant or neoadjuvant therapy was eligible. The coprimary endpoints were overall survival (OS) and progression-free survival (PFS). Pembrolizumab, compared to chemotherapy, yielded a longer median OS (10.3 vs 7.4 months) and a lower rate of treatment-related adverse events (60.9 vs 90.2%). No statistically significant differences were found in terms of PFS. Of note, long-term results with > 2 years of follow-up were consistent with those reported by Bellmunt et al*.* in [7].

The ARON project was designed to globally share and analyze real-world experiences on the use of immunotherapy in patients with genitourinary tumors. Specifically, the ARON-2 study (NCT05290038) was conducted to assess the real-world efficacy of pembrolizumab in patients recurred or progressed after platinum-based chemotherapy.

## Patients and methods

### Study population

Patients aged 18 years or older, diagnosed with advanced UC confirmed through cytological and/or histologic tests, and experiencing progression (cohort A) or recurrence (cohort B) after receiving platinum-based therapy were part of the ARON-2 study and treated with pembrolizumab between January 1, 2016, and October 1, 2022. The study involved 88 institutions from 23 countries, as detailed in Supplementary Table 1.

The entire ARON-2 dataset was analyzed in this study. To ensure data security, information was anonymized, stored in a password-protected dataset and only accessible by authorized personnel. The dataset included various patient details like age, gender, Eastern Cooperative Oncology Group-Performance Status (ECOG-PS), tumor characteristics, treatment history and response to immunotherapy. Patients lacking sufficient information on treatment response, progression or follow-up were excluded from the study.

Follow-up procedures involved regular physical examinations, laboratory tests and imaging scans (CT or MRI) at specific intervals, typically every 2–4 months, as per individual physicians' practices or when there were clinical suspicions of disease progression.

The data supporting the study's findings can be obtained from the corresponding author upon reasonable request, following ethical guidelines.

### Study endpoints

Study endpoints were determined based on the Response Evaluation Criteria in Solid Tumours version 1.1 (RECIST 1.1), categorizing disease response as complete response (CR), partial response (PR), stable disease (SD) or progressive disease (PD). The overall response rate (ORR) was calculated as the sum of CR and PR.

OS was measured from the initial administration of pembrolizumab until death, while PFS was calculated from the first pembrolizumab dose to documented disease progression or death, whichever came first. Patients without disease progression, death or lost to follow-up were censored at their last recorded visit.

### Statistical analysis

We utilized the Kaplan–Meier method with Rothman’s 95% confidence intervals (CI) to estimate OS and PFS. Comparison of survival curves was done using the log-rank test, and Cox proportional hazards models were employed to assess multivariable effects on patients’ survival and calculate hazard ratios (HRs) and 95% CIs. The Chi-square test was used for determining the difference between groups. Significance level was set at 0.05, with two-sided p values. The selection of explanatory variables of multivariate analysis was performed based on the data available in our database.

The statistical analyses were conducted using MedCalc version 19.6.4 (MedCalc Software, Broekstraat 52, 9030 Mariakerke, Belgium).

## Results

### Baseline characteristics

Eight hundred and thirty-six patients were included in our analysis. The median follow-up time was 15.3 months (95%CI 14.0 − 76.0); 613 patients (73%) were males and 223 females (27%). Median age was 71y (range 26 − 95). ECOG-PS was ≥ 2 in 143 patients (17%). Upper urinary tract carcinomas accounted for the 26% of all cases. Tumor histology was pure UC in 702 patients (84%). Variant histologies included: squamous in 64 (8%), poorly differentiated in 15 (2%), plasmacytoid in 12 (1%), neuroendocrine in 9 (1%), sarcomatoid in 7 (1%), clear cell in 6 (1%), glandular in 6 (1%), micropapillary in 5 (1%), nested in 4 (< 1%), microcystic in 2 (< 1%), lymphoepithelioma-like in 2 (< 1%) and giant cell in 2 (< 1%). Two hundred and fifty-four patients (30%) presented metastatic disease at the time of initial UC diagnosis (when in first-line). Visceral metastases were identified in 555 patients (66%).

In our analysis, 544 patients (65%) received pembrolizumab following progression to first-line platinum-based chemotherapy (cohort A) and 292 (35%) after recurring within 12 months since the completion of adjuvant or neoadjuvant chemotherapy (cohort B).

Four hundred and fifty-two (54%) were dead at time of the analysis. Treatment with pembrolizumab was ongoing in 317 patients (38%, 190 patients in cohort A, 127 in cohort B). One hundred and seventy (108 in cohort A and 62 in cohort B) of the 519 patients progressing during or after pembrolizumab treatment received further therapies. Patients’ baseline characteristics at the time of being assigned to receive pembrolizumab are summarized in Tables 1 and 2.

**Table 1:** Patients’ characteristics

	Patients (*n* = 836)
Sex	
Male	613 (73)
Female	223 (27)
Age, years (y)	
Median	71
Range	26 − 95
Interquartile ranges:	
26–63	189
64–70	229
71–76	214
77–95	204
ECOG Performance Status	
0	268 (32)
1	425 (51)
2	132 (16)
3	11 (1)
Current or former smokers	549(66)
Primary tumor location	
Upper urinary tract	220 (26)
Lower urinary tract	616 (74)
Tumor histology	
Pure urothelial carcinoma	702 (84)
Variants	134 (16)
Metastatic disease	
Synchronous	254 (30)
Metachronous	582 (70)
Common sites of metastasis	
Lymph nodes	586 (70)
Lung	280 (33)
Bone	243 (29)
Liver	152 (18)
Brain	15 (2)
Visceral metastases	
Yes	555 (66)
No	281 (34)
Patients progressed during first-line platinum-based chemotherapy (cohort A)	544 (65)
Patients relapsed within < 1y since the completion of neoadjuvant/adjuvant chemotherapy (cohort B)	292 (35)

Statistically significant values were reported in bold

ECOG-PS, Eastern Cooperative Oncology Group-Performance Status; 1y, one year

**Table 2:** Successive therapies in cohort A and B

*Successive therapies (cohort A)*	
Paclitaxel	**46 (6)**
Vinflunine	**30 (4)**
Carboplatin and Gemcitabine	**6 (1)**
Enfortumab vedotin	**6 (1)**
Clinical trials	**6 (1)**
Carboplitn and paclitaxel	**5 (1)**
Docetaxel	**4 (< 1)**
Gemcitabine	**3 (< 1)**
Carboplatin	**1 (< 1)**
MVAC	**1 (< 1)**
*Successive therapies (cohort B)*	
Vinflunine	14 (2)
Paclitaxel	12 (1)
Carboplatin and Gemcitabine	10 (1)
Clinical trials	7 (1)
Enfortumab vedotin	4 (< 1)
Gemcitabine and Palitaxel	4 (< 1)
MVAC	2 (< 1)
Cyclphosphamide	2 (< 1)
Carboplitn and paclitaxel	1 (< 1)
Gemcitabine	1 (< 1)
Carboplatin	1 (< 1)

### Survival analysis

**Fig. 1:**
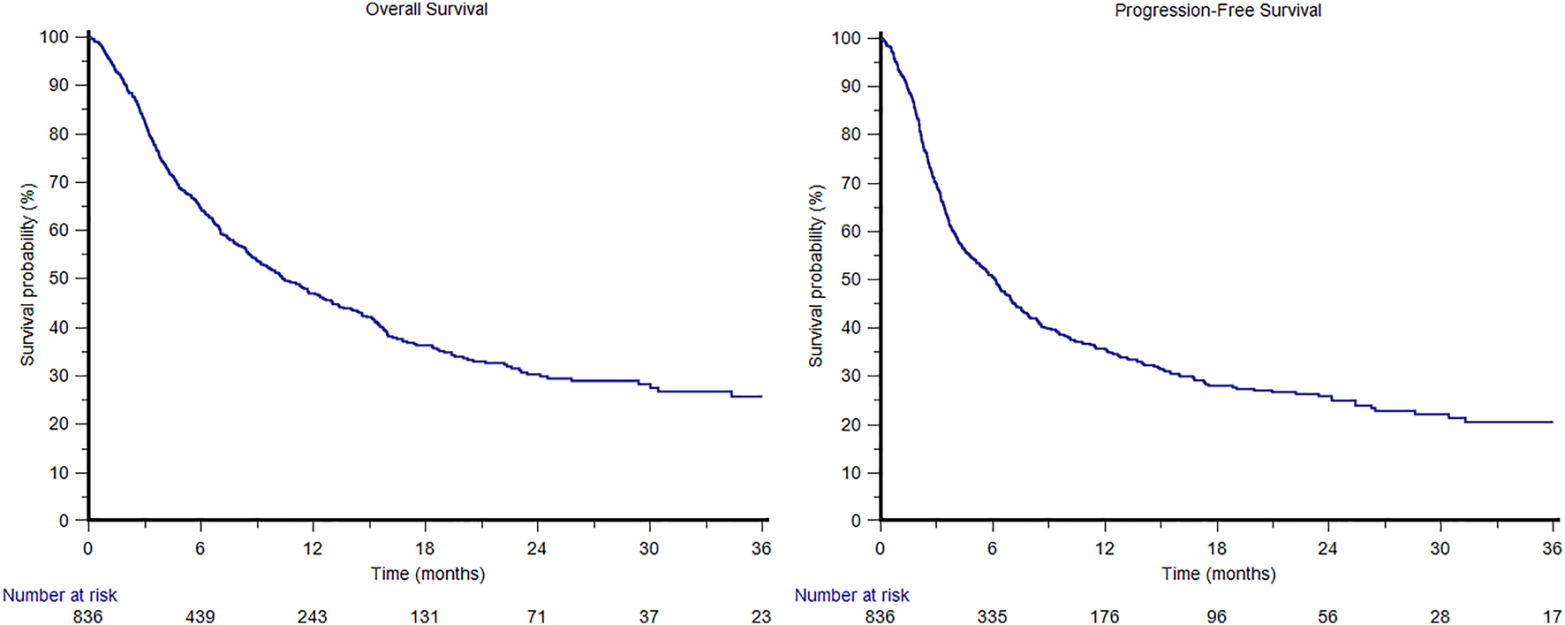
Overall and progression-free survival curves in the overall ARON-2 study population

**Fig. 2:**
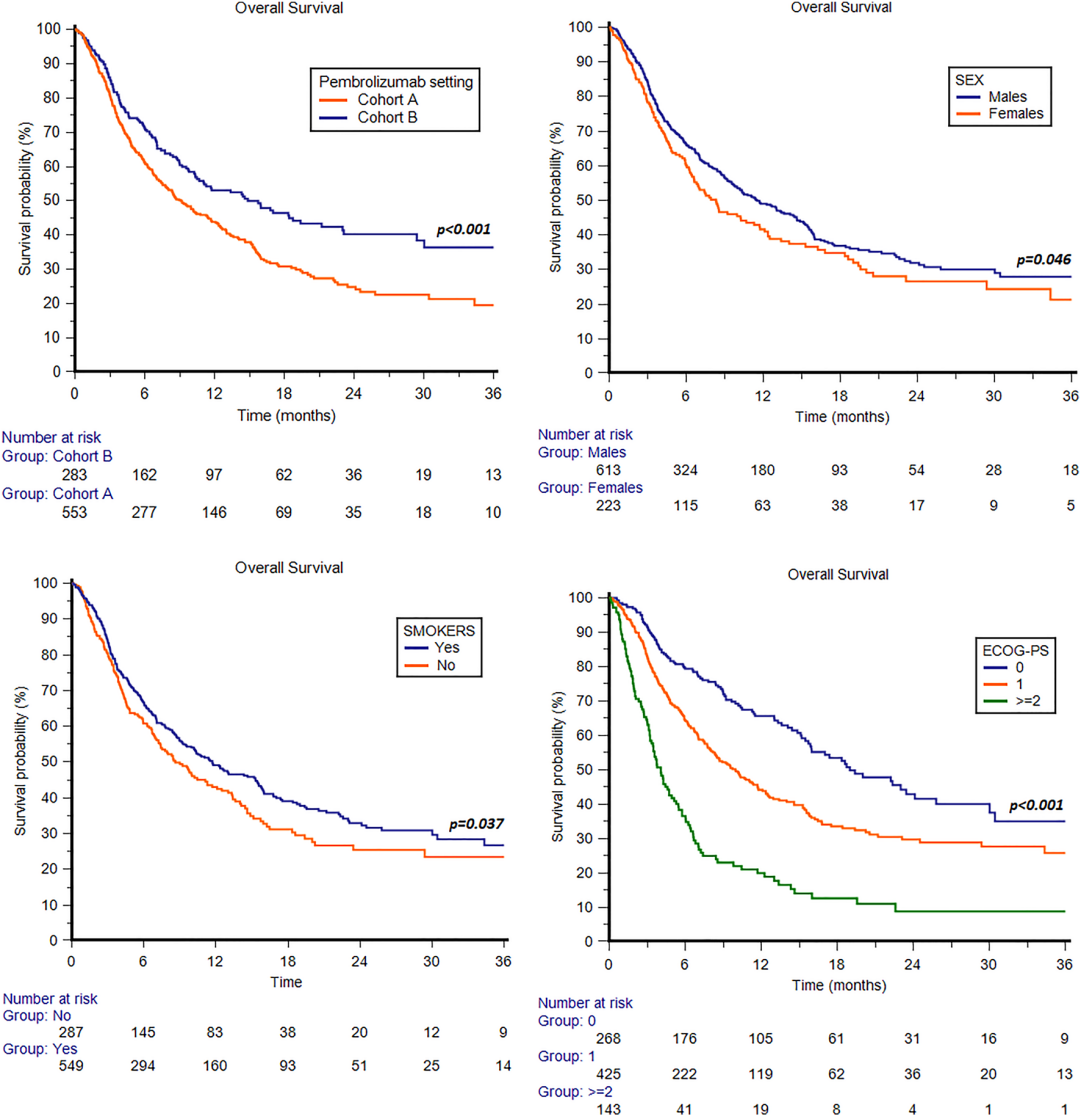
Overall survival in patients treated with pembrolizumab stratified by pembrolizumab setting (cohort A: patients progressed during first-line platinum base chemotherapy; cohort B: patients recurred within < 1y since the completion of adjuvant/neoadjuvant therapy), sex, smoking attitude and ECOG-PS

In the overall study population, the median OS was 10.5 months (95%CI 9.0 − 12.5, Fig. 1); 1-year and 2-year OS rates were 29 and 8%, respectively. The median OS was 9.1 months (95%CI 7.5 − 11.4) in cohort A and 14.6 months (95%CI 10.4 − 19.4) in cohort B (Fig. 2).

The median OS was significantly longer in males vs females (11.5 months, 95%CI 9.7 − 14.4, vs 8.3 months, 95%CI 6.4 − 11.3, *p* = 0.046, Fig. 2). Otherwise, no statistically significant differences were observed between patients aged < 65y and  ≥ 65y (10.5 months, 95%CI 8.2 − 14.9 vs 10.4 months, 95%CI 8.7 − 12.7, *p* = 0.581).

Current or former smokers showed a longer median OS compared to non-smokers (11.7 months, 95%CI 9.5 − 15.3, vs 8.6 months, 95%CI 7.0 − 11.3, *p* = 0.037, Fig. 2).

Patients stratified by ECOG-PS (0,1 or 2) showed a median OS of 19.0 months (95%CI 15.9 − 25.8), 10.0 months (95%CI 8.2 − 11.7) and 4.1 months (95%CI 3.2 − 5.1) (*p* < 0.001, Fig. 2).

Patients with pure UC histology showed a median OS of 10.8 months (95%CI 19.2 − 13.0), while in patients with mixed variant histology was 8.6 months (95%CI 6.6 − 14.6, *p* = 0.511). Interestingly, no statistically significant differences were found between patients with tumors of the upper tract (8.6 months, 95%CI 6.6 − 12.4) and lower tract (11.3 months, 95%CI 9.5 − 13.4, *p* = 0.287).

**Fig. 3:**
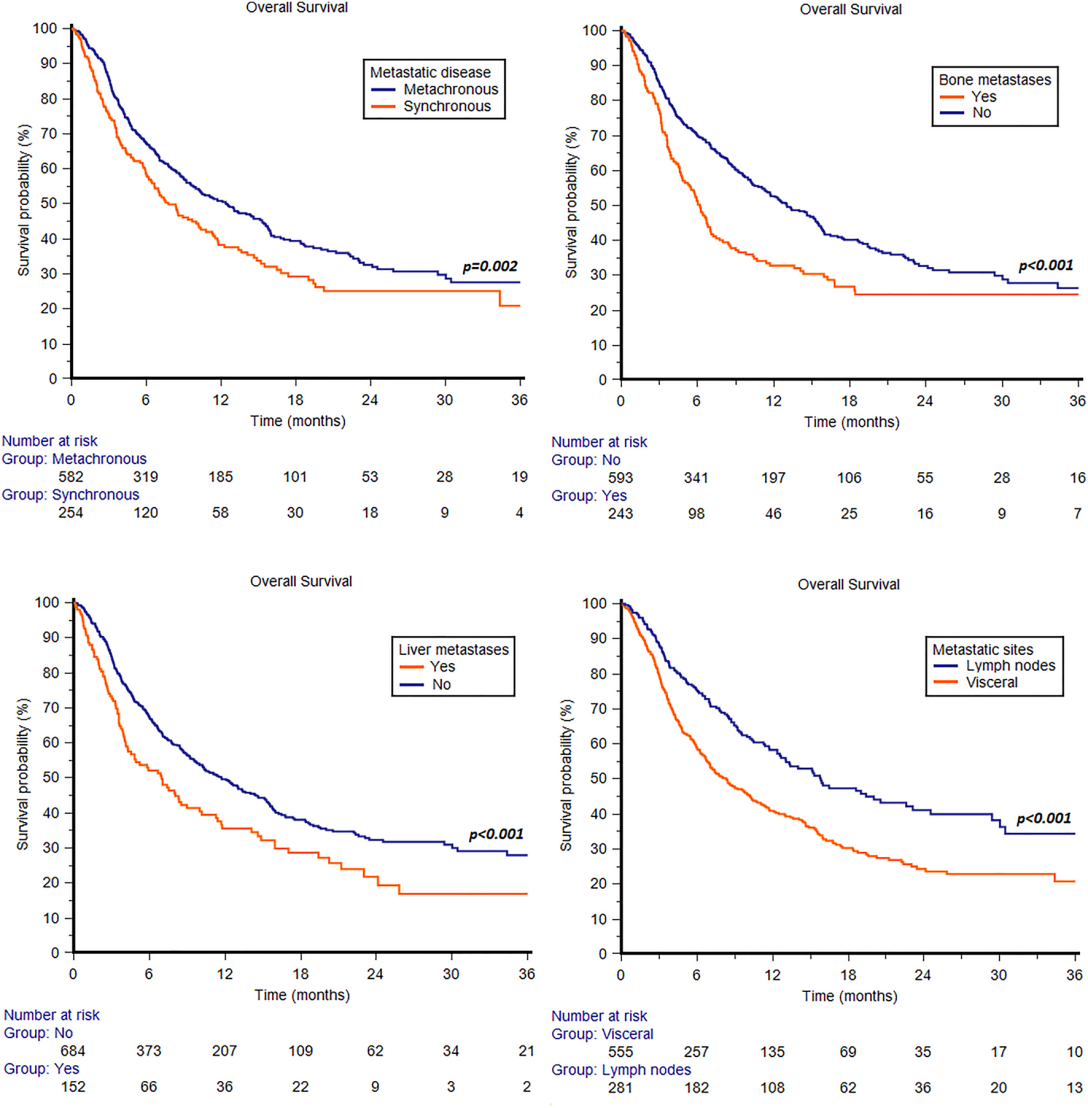
Overall survival in patients treated with pembrolizumab stratified by synchronous or metachronous metastatic disease, bone or liver metastases or visceral metastases

Synchronous metastatic disease was associated with shorter median OS (7.8 months, 95%CI 6.4 − 10.2, vs 12.5 months, 95%CI 10.0 − 15.3, *p* = 0.002, Fig. 3). By stratifying patients according to sites of metastasis, statistically significant differences were observed between patients with or without bone metastases (6.2 months, 95%CI 5.0 − 7.0, vs 13.0 months, 95%CI 11.3 − 15.4, *p* < 0.001, Fig. 3) and patients with or without liver metastases (7.0 months, 95%CI 4.3 − 9.0, vs 11.7 months, 95%CI 9.8 − 14.0, *p* < 0.001, Fig. 3). Patients with metastases confined to the lymph nodes showed longer median OS compared to those with visceral metastases (15.8 months, 95%CI 12.6 − 22.6, vs 8.4 months, 95%CI 7.0 − 10.2, *p* < 0.001, Fig. 3).

**Fig. 4:**
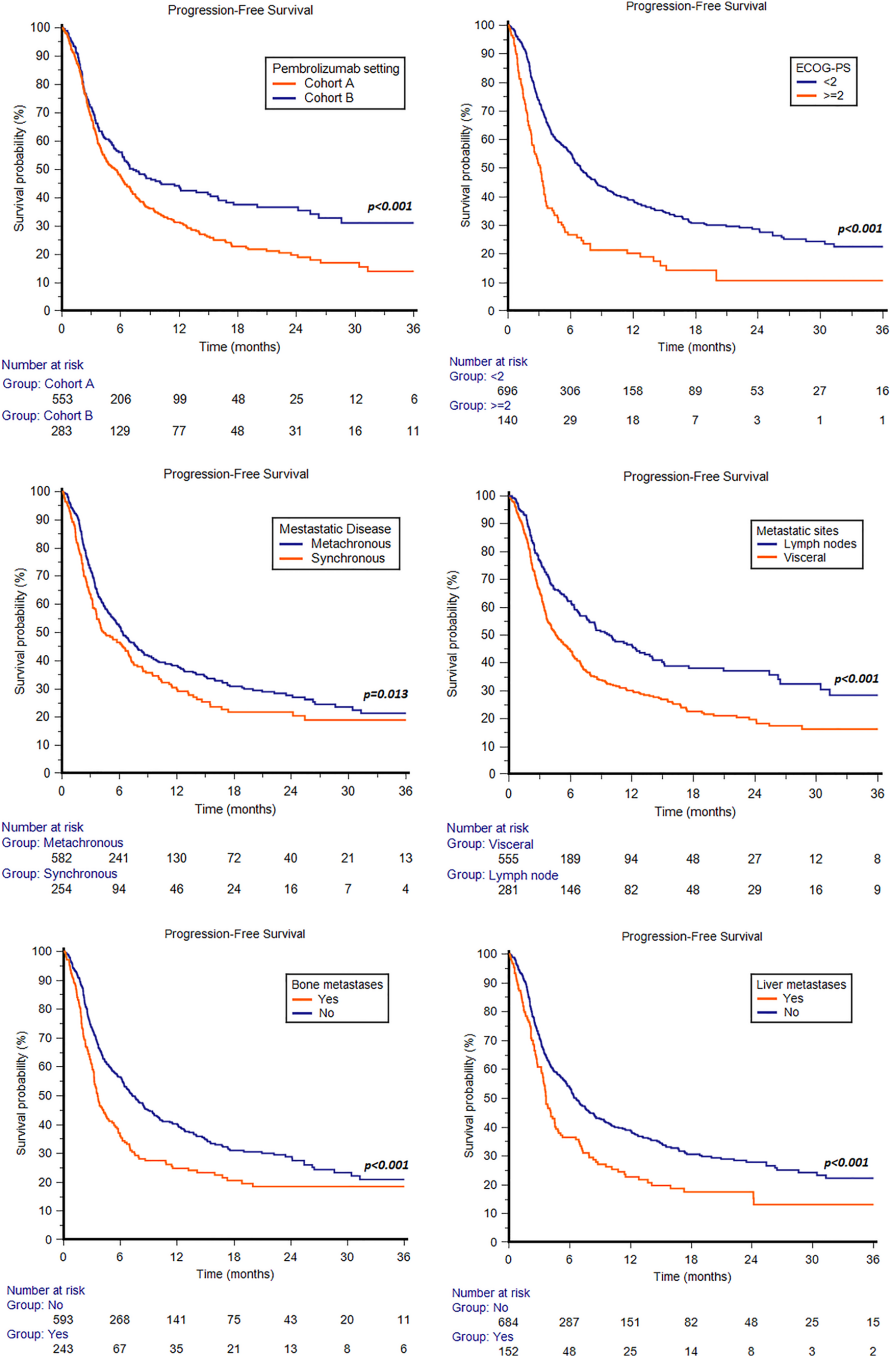
Progression-free survival in patients treated with pembrolizumab stratified by pembrolizumab setting (cohort A: patients progressed during first-line platinum base chemotherapy; cohort B: patients recurred within < 1y since the completion of adjuvant/neoadjuvant therapy), ECOG-PS, synchronous or metachronous metastatic disease, visceral metastases, bone or liver metastases

In the overall study population, the median PFS was 6.2 months (95%CI 5.1 − 6.9, Fig. 1); 1-year and 2-year PFS rates were 21 and 7%, respectively. The median PFS was 5.5 months (95%CI 4.4 − 6.4) in cohort A and 7.3 months (95%CI 5.8 − 12.0) in cohort B (*p* < 0.001, Fig. 4).

In terms of PFS, ECOG-PS ≥ 2 was associated with shorter median PFS when comparing with ECOG-PS 0–1 (3.1 months, 95%CI 2.3 − 3.5, vs 6.9 months, 95%CI 6.2 − 8.3, *p* < 0.001, Fig. 4). No statistically significant differences were observed between males and females (6.1 months, 95%CI 5.1 − 6.9 vs 6.3 months, 95%CI 4.0 − 7.9, *p* = 0.923), patients aged < 65y and ≥ 65y (6.2 months, 95%CI 4.3 − 7.5 vs 6.1 months, 95%CI 5.0 − 7.0, *p* = 0.439), smokers and non-smokers (5.5 months, 95%CI 4.2 − 6.9, vs 6.2 months, 95%CI 5.3 − 7.2, *p* = 0.441), pure and mixed UC histology (6.2 months, 95%CI 5.3 − 7.0, vs 5.0 months, 95%CI 3.9 − 44.0, *p* = 0.737), and upper and lower urinary tract (5.0 months, 95%CI 3.9 − 7.2, vs 6.3 months, 95%CI 5.4 − 7.1, *p* = 0.575).

Synchronous metastatic disease was associated with shorter median PFS compared to metachronous disease (4.4 months, 95%CI 3.6 − 6.6, vs 6.4 months, 95%CI 5.6 − 7.6, *p* = 0.013, Fig. 4). Patients with metastases confined to lymph nodes showed longer median PFS compared to those with visceral metastases (9.8 months, 95%CI 7.6 − 13.3, vs 4.6 months, 95%CI 3.9 − 5.8, *p* < 0.001, Fig. 4). By stratifying patients according to sites of metastasis, patients with bone (3.6 months, 95%CI 3.2 − 4.4, vs 7.3 months, 95%CI 6.4 − 8.6, *p* < 0.001, Fig. 4) or liver metastases (3.7 months, 95%CI 3.3 − 4.5, vs 6.6 months, 95%CI 6.0 − 7.7, *p* < 0.001, Fig. 4) showed a significantly shorter median PFS compared to patients without bone or liver metastases, respectively.

### Response to therapy

According to RECIST 1.1, 84 patients (10%) experienced CR, 179 (21%) PR, 201 (24%) SD and 372 (44%) PD, with an ORR of 31%. The median OS was significantly different according to the type of response, being NR (95%CI NR − NR), 34.4 months (95%CI 22.4 − 47.2), 15.4 months (95%CI 12.4 − 19.4) and 4.3 months (95%CI 3.8 − 30.4) in patients with CR, PR, SD and PD, respectively (*p* < 0.001).

In cohort A, CR, PR, SD and PD were registered in 6, 23, 24 and 47%. In cohort B, we observed 18% of CR, % 19% of PR, 24% of SD and 39% of PD. The difference between the ORR in the two cohorts (29% vs 37%) was not statistically significant (*p* = 0.230), although cohort B reported a higher percentage of CR (*p* = 0.009).

### Role of prognostic factors

At univariate analysis, gender, smoking attitude, ECOG-PS, synchronous metastatic disease, bone or liver metastases and pembrolizumab setting (cohort A vs cohort B) were significant predictors of OS (Table 3). As for PFS, the univariate analysis showed a prognostic role of ECOG-PS, synchronous metastatic disease, bone or liver metastases and pembrolizumab setting. At multivariate analysis, ECOG-PS ≥ 2, bone or liver metastases and pembrolizumab (cohort A vs B) proved to be significantly associated with both OS and PFS (Table 3).

**Table 3:** Univariate and multivariate analyses of predictors of overall survival and Progression-free survival in UC patients treated by pembrolizumab

Overall survival	Univariate Cox regression		Multivariate Cox regression	
	HR (95%CI)	*p-value*	HR (95%CI)	*p-value*
Gender (females vs males)	1.23(1.01 − 1.50)	**0.046**	1.21 (0.98 − 1.50)	0.080
Age (≥ 65y vs < 65y)	1.06 (0.86 − 1.31)	0.581		
Smokers vs non-smokers	0.82 (0.67 − 0.99)	**0.037**	0.87 (0.71 − 1.06)	0.166
ECOG-PS (≥ 2 vs 0–1)	2.71 (2.18 − 3.38)	** < 0.001**	2.56(2.04 − 3.20)	** < 0.001**
Histology (mixed vs pure UC)	1.08 (0.86 − 1.36)	0.511		
Upper vs Lower urinary tract	1.12 (0.91 − 1.37)	0.287		
Synchronous metastatic disease (yes vs no)	1.36 (1.12 − 1.66)	**0.002**	1.15 (0.94 − 1.41)	0.173
Lymph node (Y vs N)	0.87 (0.71 − 1.07)	0.183		
Lung metastases (Y vs N)	1.20 (0.99–1.45)	0.069		
Liver metastases (Y vs N)	1.49 (1.19 − 1.86)	** < 0.001**	1.45 (1.14 − 1.85)	**0.003**
Bone metastases (Y vs N)	1.59 (1.30 − 1.93)	** < 0.001**	1.32 (1.06 − 1.67)	**0.014**
Patients progressed during first-line therapy vs recurred within < 1y from adjuvant/neoadjuvant therapy	1.47 (1.20 − 1.80)	** < 0.001**	1.42 (1.15 − 1.75)	** < 0.001**

Progression-free survival	Univariate Cox regression		Multivariate Cox regression	
	HR (95%CI)	*p-value*	HR (95%CI)	*p-value*
Gender (females vs males)	0.99 (0.81 − 1.20)	0.923		
Age (≥ 65y vs < 65y)	0.93 (0.77 − 1.12)	0.440		
Smokers vs non-smokers	0.93 (0.78 − 1.12)	0.442		
ECOG-PS (≥ 2 vs 0–1)	2.02 (1.63 − 2.51)	** < 0.001**	1.93 (1.55 − 2.40)	** < 0.001**
Histology (mixed vs pure UC)	1.04 (0.84 − 1.29)	0.737		
Upper vs Lower urinary tract	1.06 (0.87 − 1.28)	0.576		
Synchronous metastatic disease (yes vs no)	1.26 (1.05 − 1.51)	**0.014**	1.13 (0.94 − 1.36)	0.210
Lymph node metastases (Y vs N)	0.89 (0.74 − 1.07)	0.222		
Lung metastases (Y vs N)	1.14 (0.96 − 1.37)	0.138		
Liver metastases (Y vs N)	1.52 (1.24 − 1.88)	** < 0.001**	1.43 (1.15 − 1.79)	**0.002**
Bone metastases (Y vs N)	1.52 (1.27 − 1.83)	** < 0.001**	1.27 (1.03 − 1.62)	**0.028**
Patients progressed during first-line therapy vs recurred within < 1y from adjuvant/neoadjuvant therapy	1.40 (1.15 − 1.69)	** < 0.001**	1.33 (1.10 − 1.62)	**0.003**

ECOG-PS Eastern Cooperative Oncology Group-Performance Status; UC urothelial carcinoma

Statistically significant values were reported in bold

### The ARON prognostic factors

**Fig. 5:**
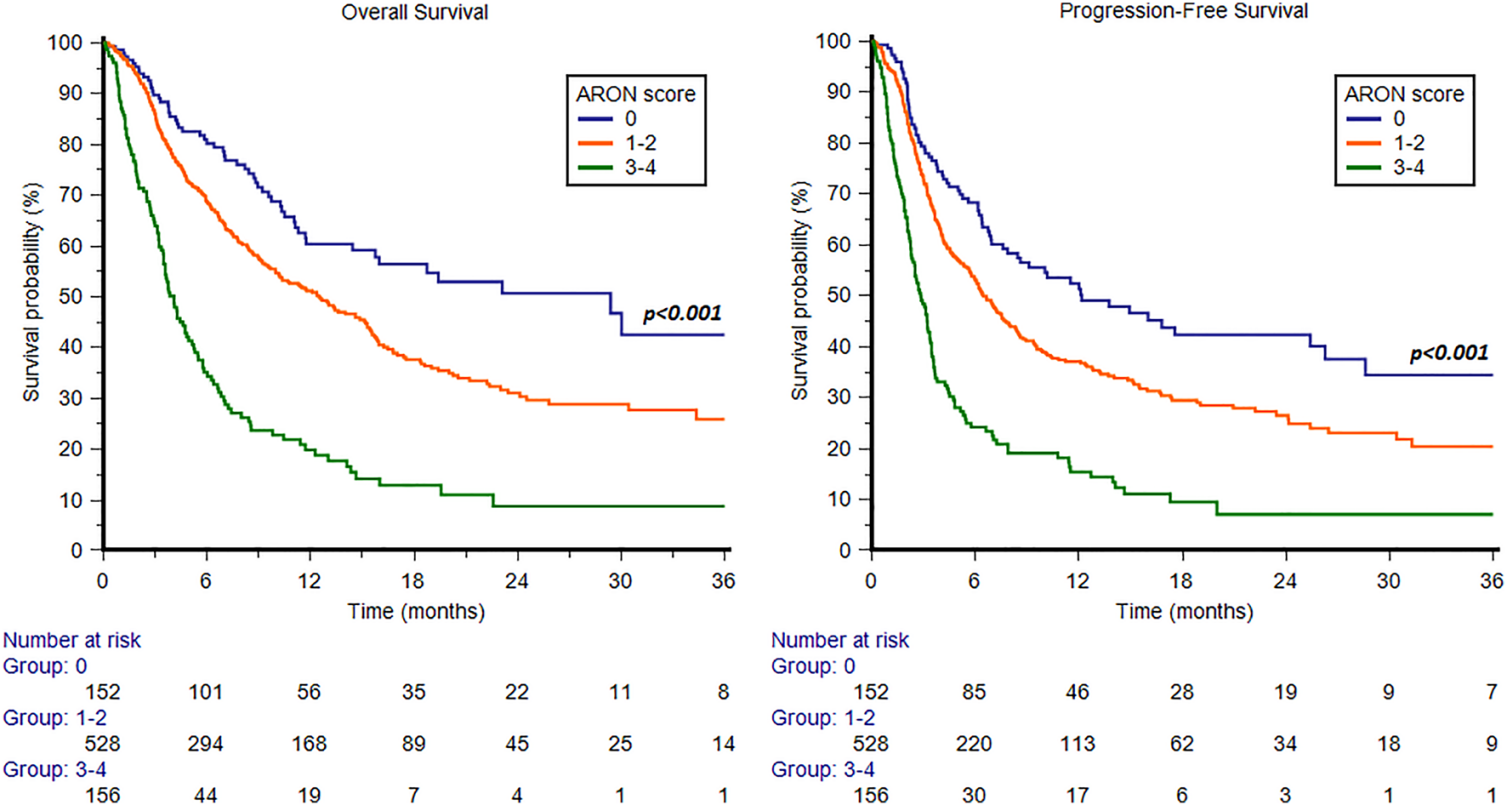
Overall survival and progression-free survival in patients stratified by ARON score

We further retrospectively analyzed patients according to the presence of the prognostic factors identified at multivariate analysis (ECOG-PS ≥ 2, bone or liver metastases and pembrolizumab pure second-line setting). We divided the study population into three groups, defined by the presence of 0, 1–2 or 3–4 prognostic factors. The median OS was 29.4 months (95%CI 14.4 − 45.9), 12.5 months (95%CI 10.0 − 15.2) and 4.1 months (95%CI 3.5 − 4.8), respectively (*p* < 0.001, c-index 0.629, 95%CI 0.596 − 0.662, Fig. 5). Accordingly, the median PFS was 12.2 months (95%CI 7.9 − 25.4), 6.4 months (95%CI 5.7 − 7.6) and 2.8 months (95%CI 2.3 − 3.4), in patients with 0, 1–2 or 3–4 prognostic factors (*p* < 0.001, c-index 0.612, 95%CI 0.578 − 0.646, Fig. 5). Furthermore, patients stratified into these 3 groups showed significantly different ORR, which was 45% in patients with 0 factors, 33% in patients with 1–2 factors and 12% in patients with 3–4 prognostic factors (*p* < 0.001). In particular, patients with 0 factors showed 23% CR, 22% PR, 26% SD and 29% PD. Patients with 1–2 factors registered 8% CR, 25% PR, 26% SD and 41% PD. On the other hand, patients with 3–4 factors reported 4% CR, 8% PR, 17% SD and 71% PD.

## Discussion

Immune checkpoint inhibitors have contributed to change the therapeutic landscape of UC [6, 7, 9, 10] and is actively developed in all therapeutic settings, [11–14]. Nevertheless, a not negligible rate of patients presents primary resistance to immune checkpoint inhibitors [15] and the majority of UC patients will present disease progression to immunotherapy. The development of validated biomarkers of response to immune checkpoint inhibitors will be crucial in order to design personalized therapeutic approaches for patients with advanced UC. To date, PD-L1 expression seems more prognostic than predictive, while it has been recently showed that tumor mutational burden (TMB) and T-cell-inflamed gene expression profile (TcellinfGEP) are associated with the outcome of patients treated with pembrolizumab in both second-line therapy and first-line therapy for cisplatn-ineligible UC patients [16].

The ARON-2 study was designed to assess the real-world efficacy of pembrolizumab in patients with advanced UC and, to the best of our knowledge, represents the largest worldwide data collection in this setting and involving 88 institutions from 23 countries it could represent an original real-world study. In this study, we focused on the second-line setting, showing that in the overall patient population, median OS and PFS were 10.5 and 6.2 months, respectively. ECOG-PS ≥ 2 and the presence of bone or liver metastases were significantly associated with worst OS and PFS, while smoking attitude was associated with longer OS, in accordance with previous studies focused on the use of immunotherapy in cancer patients [17, 18]. These findings are consistent with a recent multicenter retrospective study that included 917 patients with mUC and treated with immune checkpoint inhibitors, which reported bone and liver metastases as strong predictors of worse oncologic outcome [19]. The ORR was 31% with 10% of CR. The type of tumor response according to RECIST 1.1 criteria was a significant predictor of OS, confirming previous exploratory analysis performed in KEYNOTE-045 trial [7].

The median OS observed in the present study is very similar to the median OS reported in the pivotal KEYNOTE-045 trial (10.1 months), despite the ARON-2 population may be more representative of pembrolizumab use in real-world context. In our ARON-2 analysis, 65% of patients received pembrolizumab as second-line therapy vs 88.2% receiving pembrolizumab as second/third-line therapy in the KEYNOTE-045. Additionally, 66% had visceral metastases (18% liver) vs 89.2% (33.7% liver), and 17% had ECOG-PS ≥ 2 vs 0.7%, in ARON-2 study compared to KEYNOTE-045 trial [6, 7].

Our study presents several limitations, mainly due to its retrospective nature. A centralized review of radiological imaging was not performed and patient not assessable for response were excluded. Furthermore, we had no available data on hemoglobin levels, concomitant medications or other comorbidities that could affect the efficacy of pembrolizumab. Consequently, our results should be interpreted with caution and are in need of a larger prospective validation.

Furthermore, an important limitation related to overall survival is the low rate of successive therapies with enfortumab vedotin, which is currently the standard third-line treatment, but at the time of data collection, this treatment was not available in most of the countries enrolled in the study.

Another important point must be considered: in a Presidential Symposium at the ESMO Congress 2023 (Madrid, 20–24 October), practice-changing results were presented from the phase III trial EV-302/KEYNOTE-A39. In this trial, the combination of enfortumab vedotin with pembrolizumab almost doubled median progression-free survival (PFS) (12.5 versus 6.3 months, respectively; hazard ratio [HR] 0.45; 95% confidence interval [CI] 0.38–0.54; *p* < 0.00001) and median overall survival (OS) (31.5 versus 16.1 months, respectively; HR 0.47; 95% CI 0.38–0.58; *p* < 0.00001) compared with chemotherapy (cisplatin or carboplatin plus gemcitabine) in patients with previously untreated, locally advanced or metastatic urothelial carcinoma [20].

Nevertheless, this real-world data analysis shows that pembrolizumab was effective as second-line therapy for advanced UC patients. Further studies investigating the biological and immunological characteristics of UC patients are warranted in order to optimize the outcome of patients receiving immunotherapy in this setting.

### Supplementary information

Supplementary information

## Data Availability

The data that support the findings of this study are available from the corresponding author, F.M., upon reasonable request.
