# Supplementary material for: Global real-world experiences with pembrolizumab in advanced urothelial carcinoma after platinum-based chemotherapy: the ARON-2 study
Source: Cancer Immunol Immunother. 2024 Apr 18;73(6):106. doi: 10.1007/s00262-024-03682-w (PMC11026312; doi:10.1007/s00262-024-03682-w)
Supplement: Supplementary file 1 — Supplementary information [file 262_2024_3682_MOESM1_ESM.docx]

**Supplementary Material**

**Supplementary Table 1.** List of Countries participating to the ARON-2 study.

| **List of Countries** | | |
| --- | --- | --- |
| Argentina | Austria | Belgium |
| Brazil | Colombia | Czeck Republic |
| Germany | Greece | Guatemala |
| Hungary | Italy | Japan |
| Mexico | Morocco | Poland |
| Romania | Serbia | Singapore |
| Spain | Turkey | United Arab Emirated |
| United Kingdom | United States |  |
